# Musical activity in a subsample of the German National Cohort study

**DOI:** 10.1038/s41598-024-64773-3

**Published:** 2024-06-18

**Authors:** Juliane Menzel, Gunter Kreutz, Hans-Christian Jabusch, Heiko Becher, Lilian Krist, Thomas Keil, Friederike Borngräber, Alexander Schmidt, Stefan N. Willich, Isabel Fernholz, Cornelia Weikert

**Affiliations:** 1 Institute of Social Medicine, Epidemiology and Health Economics, Charité-Universitätsmedizin Berlin, Corporate Member of Freie Universität Berlin and Humboldt-Universität zu Berlin, Berlin, Germany; 2 Department of Food Safety, German Federal Institute for Risk Assessment, Berlin, Germany; 3 Institut für Musik, Carl von Ossietzky Universität Oldenburg, Oldenburg, Lower Saxony Germany; 4 Institute of Musicians’ Medicine, University of Music Carl Maria von Weber, Dresden, Saxony Germany; 5 Institute of Global Health, University Hospital Heidelberg, Heidelberg, Baden-Württemberg Germany; 6 Department of Neurology, Charité – Universitätsmedizin Berlin, Corporate member of Freie Universität Berlin and Humboldt Universität zu Berlin, Berlin, Germany; 7 Berlin Center for Musicians Medicine (BCMM), Charité – Universitätsmedizin Berlin, Corporate member of Freie Universität Berlin and Humboldt Universität zu Berlin, Berlin, Germany; 8 Kurt Singer Institute for Music Physiology and Musicians’ Health, Hanns Eisler School of Music Berlin and University of the Arts Berlin, Berlin, Germany; 9 Clinic for Audiology and Phoniatrics, Charité – Universitätsmedizin Berlin, Corporate member of Freie Universität Berlin and Humboldt Universität zu Berlin, Berlin, Germany; 10 Department of Psychosomatic Medicine, Charité – Universitätsmedizin Berlin, Corporate Member of Freie Universität Berlin and Humboldt Universität zu Berlin, Berlin, Germany

**Keywords:** Human behaviour, Risk factors

## Abstract

Musical activities (MA) such as singing, playing instruments, and listening to music may be associated with health benefits. However, evidence from epidemiological studies is still limited. This study aims at describing the relation between MA and both sociodemographic and health-related factors in a cross-sectional approach. A total of 6717 adults (50.3% women, 49.7% men, median age: 51 years (IQR 43–60) were recruited from the study center Berlin-Mitte of the German National Cohort (NAKO), a population-based prospective study. This study is based on a sample randomly selected from the population registry of Berlin, Germany, aged 20 to 69 years. 53% of the participants had been musically active at least once in their life (56.1% women, 43.9% men). Playing keyboard instruments (30%) and singing (21%) were the most frequent MA. Participants listened to music in median 90 min per day (IQR 30.0–150.0). Musically active individuals were more likely to have a higher education, higher alcohol consumption, were less likely to be physically active, and had a lower BMI compared to musically inactive individuals. This large population-based study offers a comprehensive description of demographic, health, and lifestyle characteristics associated with MA. Our findings may aid in assessing long-term health consequences of MA.

## Introduction

Previous research suggests that musical activities such as singing, playing musical instruments or dancing might have health benefits, especially in amateur musicians without extensive musical training^1^. The German Musikinformationszentrum (MIZ [center for music information]) reports there are 14, 3 million amateur musicians (19% of the population^2^). Therefore, the question arises whether musical activity (MA) might be considered as a health-promoting factor in the general population^3^. Epidemiological studies have discovered associations between cultural activities and health-related variables in the general population and with some emphasis on mortality^4^. For example, Bygren et al. reported a 57% increased mortality risk in Swedish adults in a cohort formed in 1991 and followed up in 2003 (n = 9011) with low cultural attendance as measured by going to concerts, participating in choral singing, and other cultural activities after adjustment for age, sex, long-term disease, income, educational level, smoking and physical exercise^5^. Løkken et al. conducted a data analysis based on the Norwegian HUNT3 study (n = 128,000; 2006–2008) suggesting that participating frequently in music, singing and theatre attendance increased self-rated health for women and reduced all-cause mortality for men^6^. However, with the inclusion of other leisure activities, the hazard ratio dropped sharply, leaving the possibility that creative leisure activities, in general, could be a protective factor. In addition, a Finnish prospective study among nearly 8000 industrial employees, who completed a questionnaire in 1986 showed that cultural activity implied a significantly reduced all-cause mortality and a reduced specific risk of cardiovascular mortality in follow-up analyses of their health records between 1986 and 2004^7^. These associations prevailed when demographic variables, socio-economic status, work stress, and health issues including diabetes and hypertension were taken into account. These studies assessed MA as part of cultural participation, which precludes any inference about the specific contributions as compared to other cultural practices, leisure activities, or hobbies.

A review of studies that could have implications for public health, but were drawn from (quasi-)experimental approaches and intervention studies, showed that MA might entail both physical and mental health benefits^8^. For example, it has been shown that singing and playing an instrument can have favorable influences on the human stress response, which, in turn can affect both physical and mental wellbeing^9,10^. Such observations are partially mirrored also in cohort studies, which have begun to consider singing and playing musical instruments as a categorical variable. Specifically, Ekholm et al. analyzed data from the representative Danish Health and Morbidity Survey 2013 (n = 25,000 adults) and found that 31.8% of musically active individuals expressed strong belief in positive health consequences^11^. Moreover, such belief was greater in women and in individuals who played musical instruments or sung for an hour per day on average as compared to musically less engaged individuals. Finally, empirical studies in naturalistic settings suggest that MA can enhance social wellbeing^12,13^, reduce loneliness and thus may be beneficial for mental health, for example, by enhancing morale and reducing risk of depression in older adults^9^. In sum, these different lines of work align in their observations that when MA is considered independently, relevant health consequences can be noted, even when the diverging characteristics associated with singing as opposed to learning to play and performing on musical instruments are disregarded.

MA has also been discussed as a protective factor in dementia^14,15^, with frequently playing musical instruments and dancing being associated with low hazard ratios (0.31 and 0.24, respectively). Although the mechanism driving such effects are unclear, MA could be beneficial due to frequently observed favorable stress and immune responses^16–18^. Findings from a range of experimental and field studies suggest beneficial effects of MA on physical and psychological wellbeing^19^. In addition, there is ongoing research on health-promoting effects of listening to music by reducing stress^20^. Despite uncertainties about the mechanisms which drive such effects, there is consensus of influences of the individual musical biography, preferences, and interests in music^21^. Recently, effects of listening to music on emotion regulation during the covid-19 pandemic were confirmed in several studies^22–24^.

Despite the growing evidence of potential health effects of MA, there is still a lack of cohort studies that allow a more detailed analysis of associations between musical engagement and health outcomes^25^. One reason was probably the lack of a survey instrument, but with the development of the MusA questionnaire (questionnaire about MA) a suitable tool is now available^3^. As a prelude, this study aimed to present cross-sectional results of the MusA questionnaire using data from the NAKO study center Berlin-Mitte. This baseline paper presents differences in basic characteristics, as well as sociodemographic and health-related factors in participants with different levels of active and receptive music activities.

## Methods

### Study population

The present study used data from the German National Cohort (NAKO), a population-based, prospective study that enrolled more than 200,000 participants in 18 study centers across Germany with the aim of identifying causes of common chronic diseases and detecting their preclinical stages^26^. Participants were between 20 and 69 years of age at the timepoint they were included in the study^27^. The study design of the NAKO has been described in detail elsewhere^26,28^. NAKO was approved by the ethical review committees of all participating study centers including the Charité—Universitätsmedizin Berlin and all procedures followed the guidelines as represented in the Declaration of Helsinki in its current form. Written informed consent was obtained from all participants. As stated above, the MusA questionnaire was applied in the study center NAKO Berlin-Mitte. The baseline examination in the NAKO Berlin-Mitte took place from 2014 to 2019 (n = 11,048). The MusA questionnaire was only used from 2016 onwards. 879 participants completed the MusA questionnaire during their baseline examination (2016–2019). 5913 individuals completed the MusA questionnaire during their first follow-up examination (2019–2022). Since the present study focused on amateur musicians only, professional musicians (n = 75) were excluded from the present analyses. The total study population comprised 6717 participants (women = 3336, men = 3381).

### Assessment of musical activity

In the MusA questionnaire, participants indicated how many hours/minutes a day they listened to music in the last 12 months and which music genres they preferred. The music genres were predefined: Rock/Pop, Classical music, German Schlager, Jazz, Oldies/Evergreens, Dance/Hiphop/Rap, Hard Rock/Heavy metal, Techno/House, Folk music/Brass music, Opera/Operetta/Singing, Musicals or other. Further, MusA assessed the number of years of music making and the respective weekly practice hours in five individual life stages (≤ 10 years, 11–20 years, 21–30 years, 31–50 years, > 50 years). Participants counted as non-musically active if they had not played an instrument or sung at any stage of their life. In contrast, participants counted as musically active if they had played an instrument or sung at a single or at multiple stages of their life. We calculated the total music-hours for each life stage (years of music making multiplied by annual active music hours) based on the information about the years of music making and the weekly active music hours (conversion into annual active music hours by factor 52.14). Further, we calculated the cumulative lifetime music-hours as the sum of total music-hours of all life stages. Lifetime active music years are the summation of the years of music making of all life stages. Practice density was calculated from cumulative lifetime music-hours divided by lifetime active music years. The MusA questionnaire also assessed the main instrument at the individual peak of MA and assessed the MA of the last 12 months (main instrument, practice times etc.).

### Lifestyle factors and other variables

During baseline assessment, all study participants underwent an extensive examination program: Age, sex, and nationality were assessed via self-report. Various information about socio-economic characteristics were assessed by a standardized, computer-assisted face-to-face interview i.e., household size, living situation, education, employment status, family net income, and migration background. Education level was determined using the International Standard Classification of Education^29^, further categorized as ‘low’, ‘medium’ or ‘high’ education. The equivalized income was calculated by dividing the family net income by the number of household members (converted into equalized adults). Smoking status, alcohol consumption and physical activity were assessed via touchscreen-based self-administered questionnaires. In addition, the Alcohol Use Disorders Identification Test short version (AUDIT-C) was applied to assess hazardous drinking. Risky alcohol consumption was defined as AUDIT-C Score > 4 for men, and > 3 for women^30^. Physical activity was measured using the Global Physical Activity Questionnaire^31^. Based on this information Metabolic Equivalents (MET)-min/week were calculated. MET minutes per week indicate how much energy is expended on various activities throughout the week and are a surrogate for activity intensity. Trained study staff (according to standard operating procedures) measured weight and height when participants were wearing underwear without shoes using a calibrated integrated measurement station (SECA model 764, Seca®, Hamburg, Germany). BMI was calculated by dividing body weight (kg) by height in meters squared (m^2^). In addition, BMI was also categorized according to common BMI categories, as follows: underweight (BMI < 18.5 kg/m^2^), normal weight (18.5 ≤ BMI < 25.0 kg/m^2^), overweight (25.0 ≤ BMI < 30.0 kg/m^2^), obesity (≥ 30.0 kg/m^2^). Waist circumference was measured at the midpoint between the lower rib and the iliac crest. The subjective life satisfaction and subjective health status were assessed by questionnaires^32,33^. Likert scales were used to have the respondents rate the extent to which they agreed with their personal life satisfaction, ranging from completely unsatisfied (0) to completely satisfied (10), and health status, ranging from excellent (1) to bad (5)^32,33^.

### Statistics

Normal distribution was tested for all continuous variables. As all variables were skewed, they were reported as median and interquartile range (IQR) in all tables. Categorical variables were reported as n (percentages). The present study characterized the sample of musically active participants (amateur musicians), as well as music listeners at baseline in terms of socio-demographic data, physical activity, BMI and other lifestyle factors such as smoking and alcohol consumption. To investigate differences in basic characteristics of participants who were musically less vs. more active, a median split procedure was carried out for the cumulative lifetime music-hours (median: 1042.8 h). A Mann–Whitney U test was used to compare the continuous variables between different groups, and a chi-square test was used for categorical variables. Possible associations between music listening minutes (minutes/day) and other variables were investigated by using categorization into tertiles of music listening minutes per day (T1 (Min–Max): 0–50 min/day, T2: 60–120 min/day, T3: 125–1440 min/day). Orthogonal polynomial contrasts were used in analysis of variance (ANOVA) or multivariable adjusted analysis of covariance (ANCOVA) to test for linear trends across tertiles of music listening minutes per day. ANCOVA was only performed for cumulative lifetime music-hours, lifetime active music years and practice density, adjusted for sex, age, education, smoking, physical activity, BMI, employment status, and monthly equivalized income. As all variables were skewed, variables were log-transformed prior to ANCOVA and afterwards back-transformed. As the geometric means useful summaries skewed data, cumulative lifetime music-hours, lifetime active music years and practice density were expressed as geometric means and 95%-confidence intervals (95% CI). As extended analyses, potential predefined determinants (i.e. sex, age, education, smoking, physical activity, BMI, employment status, monthly equivalized income) of cumulative lifetime music-hours were investigated using regression analyses, examined in Model 1 (unadjusted) and further mutually adjusted in Model 2 using data of all participants (n = 6717). The exposure variable (cumulative lifetime music-hours) and all continuous determinants (i.e. age, physical activity, BMI, monthly equivalized income) were log-transformed prior to the analyses. All statistical analyses were performed using SAS software, version 9.4 (SAS Institute, Cary, NC, USA). P-values were not corrected for multiple testing.

## Results

Table 1 shows the basic characteristics of the total study population (n = 6717) with a median of 51 years of age (IQR 43.0–60.0). In the last 12 months, 23.5% were musically active. A total of 53% (n = 3512) of the study population were musically active across one or more stages of life, whereas 3109 individuals were never musically active (N = 6621, missing information n = 96). Musically active and inactive groups were found similar with respect to their age ranges. Table 1 also shows that MA was more frequent in females as compared to males. Indeed 70.9% of singers and a similar majority of woodwind players were female, who also dominated keyboard playing with 58.6%. By contrast, plucked, brass and percussion instruments were more male-dominated, leaving strings as the only category with a balanced sex distribution (see Supplemental Table 1, for details). With 98.6% indicating Caucasian descent, 15.9% also indicated a migration background. The latter proportion is lower than the total proportion in the entire city of Berlin, which amounts to 24.3%^34^. The socioeconomic variables revealed an advantage of the MA group with respect to both education and family income (Table 1). Accordingly, no differences have been seen in sex-stratified analyses (Supplemental Table 2). Individuals in the MA group were more likely to work part time (applies mainly to men, Supplemental Table 2) and to live in households of more than three people (Table 1). Overall, with 69.8% full employment and an unemployment rate of 3.1% in our total study sample, their unemployment rate was lower than that of the entire city of Berlin, where the current statistics indicates an unemployment rate of more than 9%^35^.

**Table 1:** Basic characteristics of the total study population and according to musical activity.

	Total population (n = 6717)		No musical activity (n = 3109)^ac^		Musically active (n = 3512)^bc^	
	% (n) or median (IQR)	Missing % (n)	% (n) or median (IQR)	Missing % (n)	% (n) or median (IQR)	Missing % (n)
Age (years)	51.0 (43.0–60.0)		53.0 (45.0–62.0)		49.0 (41.0–58.0)	
Sex						
Men	49.7% (3336)		56.3% (1750)		43.9% (1542)	
Women	50.3% (3381)		43.7% (1359)		56.1% (1970)	
Lifestyle						
Smoking status		2.0% (134)		2.2% (69)		1.6% (57)
Non-smoker	44.6% (2935)		45.5% (1383)		43.7% (1511)	
Ex-smoker	33.6% (2215)		32.2% (979)		34.9% (1207)	
Smoker	21.8% (1433)		22.3% (678)		21.4% (737)	
Alcohol consumption (g/day)						
Men	9.2 (2.8–20.2)	1.1% (71)	8.4 (2.4–20.3)	1.3% (41)	9.6 (3.2–19.8)	0.7% (25)
Women	3.9 (1.1–10.6)	1.4% (95)	3.3 (0.8–9.5)	1.4% (45)	4.4 (1.3–11.9)	1.3% (46)
Risky alcohol consumption^d^	40.5% (3917)	2.1% (138)	38.1% (1156)	2.3% (71)	42.5% (1467)	1.7% (60)
Physical activity (MET-min/week)	3640 (1680–8280)	5.9% (396)	4080 (1680–9329)	6.2% (192)	3360 (1680–7080)	5.8% (202)
BMI (kg/m^2^)	24.9 (22.4–27.9)	0.2% (13)	25.6 (23.1–28.8)	0.2% (5)	24.3 (21.9–27.0)	0.2% (6)
Underweight (< 18.5 kg/m^2^)	1.2% (82)	0.2% (13)	0.7% (22)	0.2% (5)	1.7% (60)	0.2% (6)
Normal weight (18.5 ≤ to < 25.0 kg/m^2^)	49.0% (3286)		42.4% (1315)		55.4% (1942)	
Overweight (≥ 25.0 to < 30.0 kg/m^2^)	34.8% (2334)		37.8% (1175)		31.7% (1111)	
Obesity (≥ 30.0 kg/m^2^)	15.0% (1002)		19.1% (592)		11.2% (393)	
Waist circumference (cm)						
Men	93.0 (85.2–101.0)	0.2% (16)	94.8 (86.5–103.4)	0.4% (11)	90.8 (83.5–98.2)	0.1% (4)
Women	81.0 (74.0–89.6)	0.3% (22)	83.0 (75.2–92.2)	0.2% (6)	79.7 (73.2–88.0)	0.4% (15)
Monthly equivalized income (€)	2033 (1433–2833)	4.5% (300)	2033 (1400–2750)	4.3% (135)	2100 (1433–2875)	4.5% (159)
Education		10.5% (702)		9.1% (284)		11.7% (412)
High	70.1% (4214)		60.9% (1721)		79.0% (2449)	
Medium	28.4% (1708)		37.0% (1044)		20.3% (631)	
Low	1.5% (93)		2.1% (60)		0.7% (20)	
Household size		0.0% (1)				0.1% (1)
Single person household	25.8% (1730)		25.9% (805)		25.6% (898)	
Household with 2 persons	45.1% (3030)		49.0% (1522)		41.6% (1462)	
Household more than 3 persons	29.1% (1956)		25.1% (782)		32.8% (1151)	
Living situation		0.1% (6)		0.1% (2)		0.1% (4)
Apartment	84.6% (5678)		82.5% (2562)		86.5% (3034)	
House	15.3% (1028)		17.4% (542)		13.4% (472)	
Assisted living	0.1% (5)		0.1% (3)		0.1% (2)	
Employment status		1.0% (64)		1.2% (38)		0.7% (26)
Employment	78.9% (5247)		75.1% (2308)		82.6% (2881)	
Unemployed	3.1% (211)		3.4% (104)		2.8% (97)	
Retired	18.0% (1195)		21.5% (659)		14.6% (508)	
Extent of employment		24.1% (1622)		27.7% (862)		20.5% (720)
Full-time (≥ 35 h/week)	69.8% (3558)		73.9% (1660)		66.7% (1862)	
Part-time (< 35 h/week)	30.2% (1537)		26.1% (587)		33.3% (930)	
Other						
Subjective life satisfaction		2.3% (155)		2.6% (80)		1.9% (68)
9 to completely satisfied (10)	35.6% (2337)		38.2% (1156)		33.3% (1145)	
6 to 8	52.0% (3414)		50.1% (1517)		54.0% (1861)	
5	5.8% (379)		5.5% (166)		5.8% (203)	
2 to 4	5.7% (374)		5.2% (161)		6.1% (209)	
Completely unsatisfied (0) to 1	0.9% (58)		1.0% (29)		0.8% (26)	
Subjective health status		2.3% (155)		2.6% (80)		1.9% (68)
Excellent	4.7% (310)		3.8% (118)		5.4% (187)	
Very good	34.3% (2253)		31.0% (938)		37.8% (1301)	
Good	52.4% (3435)		55.3% (1675)		49.5% (1703)	
Less good	7.9% (517)		9.0% (271)		6.8% (235)	
Bad	0.7% (47)		0.9% (27)		0.5% (18)	

Data expressed as median (IQR) or percentage (n).

^a^Participants count as non-musically active if they have not played an instrument or sung at any stage of life.

^b^Participants count as musically active if they have played an instrument or sung at a single or multiple stages of life.

^c^Missing information on musical activity (n = 96).

^d^Risky alcohol consumption was defined as AUDIT-C Score > 4 for men, and > 3 for women.

Concerning lifestyle variables, smoking was similar across groups, but MA individuals were in median less physically active, showed lower BMI and waist circumference values, but consumed more alcohol than their musically inactive peers (Table 1). Moreover, even after adjusting for age and sex MA individuals showed a lower BMI compared to the musically inactive group (data not shown). Further, basic characteristics according to musical activity stratified sex were presented in Supplemental Table 2.

### Musical activity

**Figure 1:**
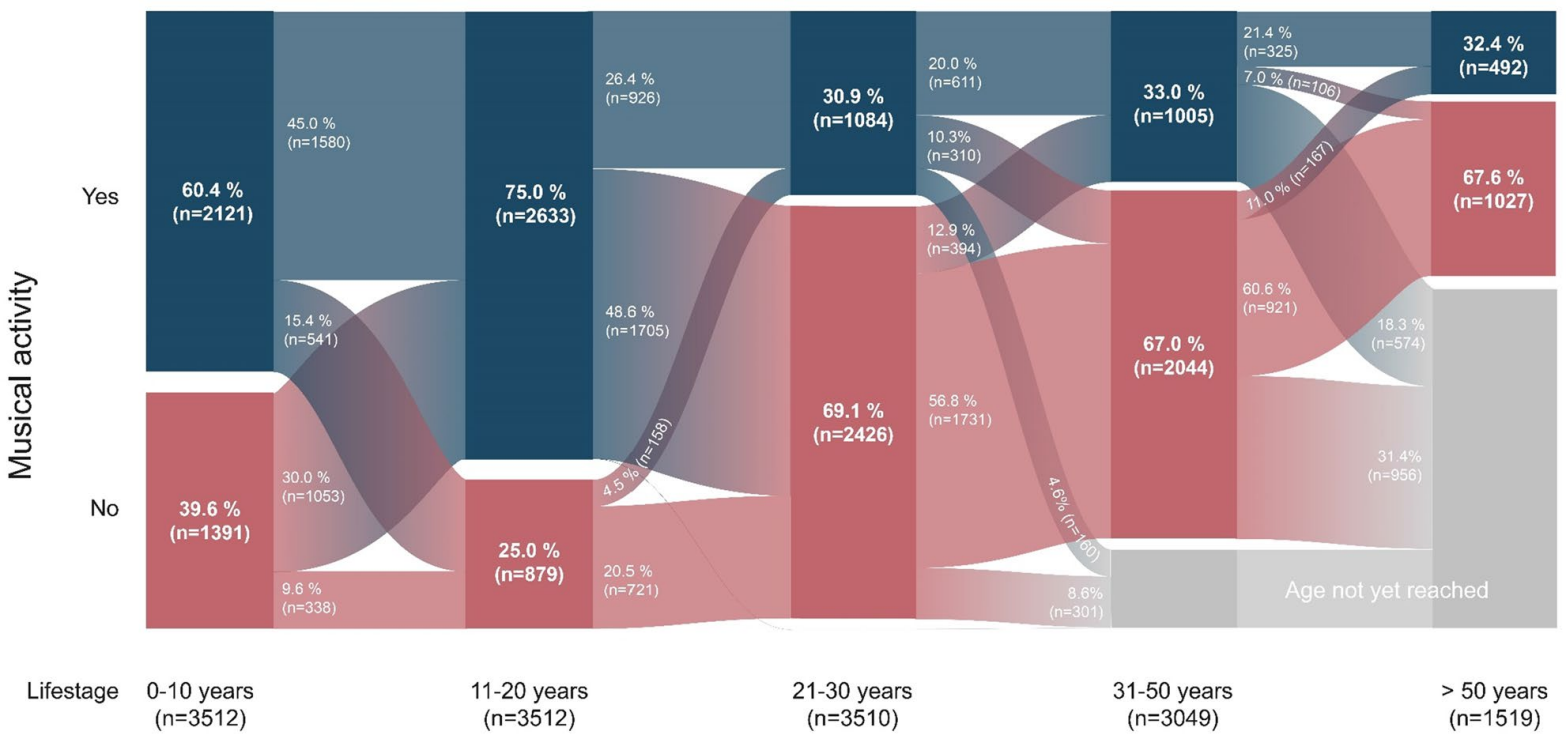
Percentage of musical activity across different life stages (musically active study population n = 3512). Results are given for musically active persons (n = 3512) only, musically inactive persons are not considered (n = 3109). Blue boxes display participants who were musically active in the respective life stages. Red boxes display participants who were not musically active in the respective life stages. Blue- or red-fading streams indicate changes of musical activity between different life spans of participants. A stream from blue to red indicates a change from a musically active to a musically inactive life stage. A stream from red to blue indicates a change from a musically inactive to musically active life stage. Grey-fading streams display an age restriction of study participants which were younger than the considered life stage.

Considering MA across one or more stages of life we observed that 60.4% (n = 2121) of all musically active individuals (n = 3512) commenced this activity in their early childhood (≤ 10 years of age). Figure 1 shows that the highest percentage of MA was seen between 11 and 20 years of age (75.0%, n = 2633). MA is present in all adult age groups (21–30 years: n = 1084; 31–50 years: n = 1005; > 50 years: n = 492). In total, 476 individuals reported continued MA for their entire life (participants age ≤ 20 years: n = 1; ≤ 30 years: n = 107; ≤ 50: n = 230; > 50 years: n = 138).

Supplemental Table 3 presents the extent (years of music making) and active music hours (hours/week) of musical activities in individual life stages. Active music hours per week differed only slightly across individual life stages (median 2 to 3 h/week). To combine the information of extent and weekly active music hours the present study focused on the cumulative lifetime music-hours (n = 3121, missing information n = 391). In median, the cumulative lifetime music-hours in musically active participants accounted for 1042.8 h (IQR 417.1–2659.1). The musically active study population (i.e. instrumentalists and singers) had a median of 7.0 lifetime active music years (IQR 4.0–14.0), corresponding to a median practice density of 156.4 h per active music year (IQR 104.3–231.2). In the total population (N = 6717) the main determinants in individuals with high cumulative lifetime music-hours in the unadjusted model 1 (Supplemental Table 4) were female sex, lower age, lower physical activity, low BMI, high monthly equivalized income, high education and employment. Only slight variations occurred in the mutually adjustment model (Supplemental Table 5).

### Musical activity and lifestyle

Table 2 presents a comparison between musically less active vs. more active participants by means of a median split of cumulative lifetime music-hours (< 1042.8 h vs. ≥ 1042.8 h). The former group accumulated only 3 years of playing music/singing in median in their life course and had about half of the practice density compared to the more active group, i.e. hours of practice per active year of singing or playing an instrument (Table 2). We observed no differences between the musically less active and the more active group according to sex, age, or any other lifestyle factor, as well as household size living situation (all p ≥ 0.05, Table 2). However, the musically more active group had a higher education status and was more likely to work part-time (< 35 h/week). In addition, the musically more active group tended to have a better subjective health status and subjective life satisfaction (Table 2).

**Table 2:** Differences between musically less active and more active participants (assessed by cumulative lifetime music-hours median split) (n = 3121, missing information n = 391).

		Cumulative lifetime music-hours		p-value
		< 1042.8 h (n = 1471)	≥ 1042.8 h (n = 1650)	
Years of making music (years)	Median (IQR)	3.0 (2.0–5.0)	13.0 (8.0–23.0)	< 0.0001
Practice density (hours/active music year)	Median (IQR)	104.3 (56.9–156.4)	206.6 (150.6–312.8)	< 0.0001
Musical activity for entire life	Percentage (n)	2.2% (33)	25.9% (427)	
Age (years)	Median (IQR)	48.0 (40.0–57.0)	48.0 (41.0–56.0)	0.64
Sex				0.55
Men	Percentage (n)	43.7% (643)	44.8% (739)	
Women	Percentage (n)	56.3% (828)	55.2% (911)	
Lifestyle				
Smoking status				0.15
Non-smoker	Percentage (n)	44.9% (650)	43.6% (708)	
Ex-smoker	Percentage (n)	33.0% (478)	36.2% (588)	
Smoker	Percentage (n)	22.1% (320)	20.2% (328)	
Alcohol consumption (g/day)				
Men	Median (IQR)	9.5 (3.3–18.5)	10.4 (3.2–20.0)	0.25
Women	Median (IQR)	4.1 (1.4–10.9)	4.5 (1.3–11.9)	0.93
Risky alcohol consumption^a^		41.0% (594)	44.5% (722)	0.05
Physical activity (MET-min/week)	Median (IQR)	3280 (1600–2833)	3360 (1680–7200)	0.31
BMI (kg/m^2^)	Median (IQR)	24.1 (21.9–27.0)	24.2 (21.9–26.8)	0.49
Underweight (< 18.5 kg/m^2^)	Percentage (n)	1.6% (23)	1.9% (31)	0.90
Normal weight (18.5 ≤ to < 25.0 kg/m^2^)	Percentage (n)	56.7% (831)	57.0% (939)	
Overweight (≥ 25.0 to < 30.0 kg/m^2^)	Percentage (n)	30.8% (453)	30.2% (498)	
Obesity (≥ 30.0 kg/m^2^)	Percentage (n)	10.9% (160)	10.9% (180)	
Waist circumference (cm)				
Men	Median (IQR)	90.0 (82.8–97.8)	90.3 (83.8–97.2)	0.57
Women	Median (IQR)	79.2 (73.1–87.6)	79.1 (73.0–87.7)	0.66
Monthly equivalized income (€)	Median (IQR)	2125 (1433–2833)	2111 (1433–2969)	0.89
Education				0.0005
High	Percentage (n)	76.8% (978)	82.6% (1223)	
Medium	Percentage (n)	22.6% (288)	16.8% (248)	
Low	Percentage (n)	0.6% (7)	0.6% (9)	
Household size				0.88
Single person household	Percentage (n)	25.6% (376)	25.0% (412)	
Household with 2 persons	Percentage (n)	40.7% (589)	41.5% (685)	
Household more than 3 persons	Percentage (n)	33.7% (496)	33.5% (553)	
Living situation				0.33
Apartment	Percentage (n)	86.0% (915)	87.4% (1440)	
House	Percentage (n)	14.0% (205)	12.6% (207)	
Assisted living	Percentage (n)	0.0% (0)	0.0% (0)	
Employment status				0.20
Employment	Percentage (n)	84.2% (1228)	83.3% (1366)	
Unemployed	Percentage (n)	2.1% (31)	3.2% (52)	
Retired	Percentage (n)	13.7% (200)	13.5% (221)	
Extent of employment				0.02
Full-time (≥ 35 h/week)	Percentage (n)	68.8% (825)	64.5% (850)	
Part-time (< 35 h/week)	Percentage (n)	31.2% (374)	35.5% (467)	
Other				
Subjective life satisfaction				0.03
9 to completely satisfied (10)	Percentage (n)	30.2% (436)	34.8% (564)	
6 to 8	Percentage (n)	56.0% (807)	54.0% (875)	
5	Percentage (n)	6.2% (90)	5.5% (89)	
2 to 4	Percentage (n)	6.7% (96)	5.2% (85)	
Completely unsatisfied (0) to 1	Percentage (n)	0.9% (13)	0.5% (8)	
Subjective health status				0.03
Excellent	Percentage (n)	5.1% (73)	6.1% (98)	
Very good	Percentage (n)	35.9% (518)	40.6% (659)	
Good	Percentage (n)	51.3% (743)	46.9% (760)	
Less good	Percentage (n)	6.9% (99)	5.8% (95)	
Bad	Percentage (n)	0.6% (9)	0.6% (9)	

Data expressed as median (interquartile range (IQR)) or percentage (n), Group comparisons were calculated by chi-square tests or Mann–Whitney U tests.

^a^Risky alcohol consumption was defined as AUDIT-C Score > 4 for men, and > 3 for women.

**Figure 2:**
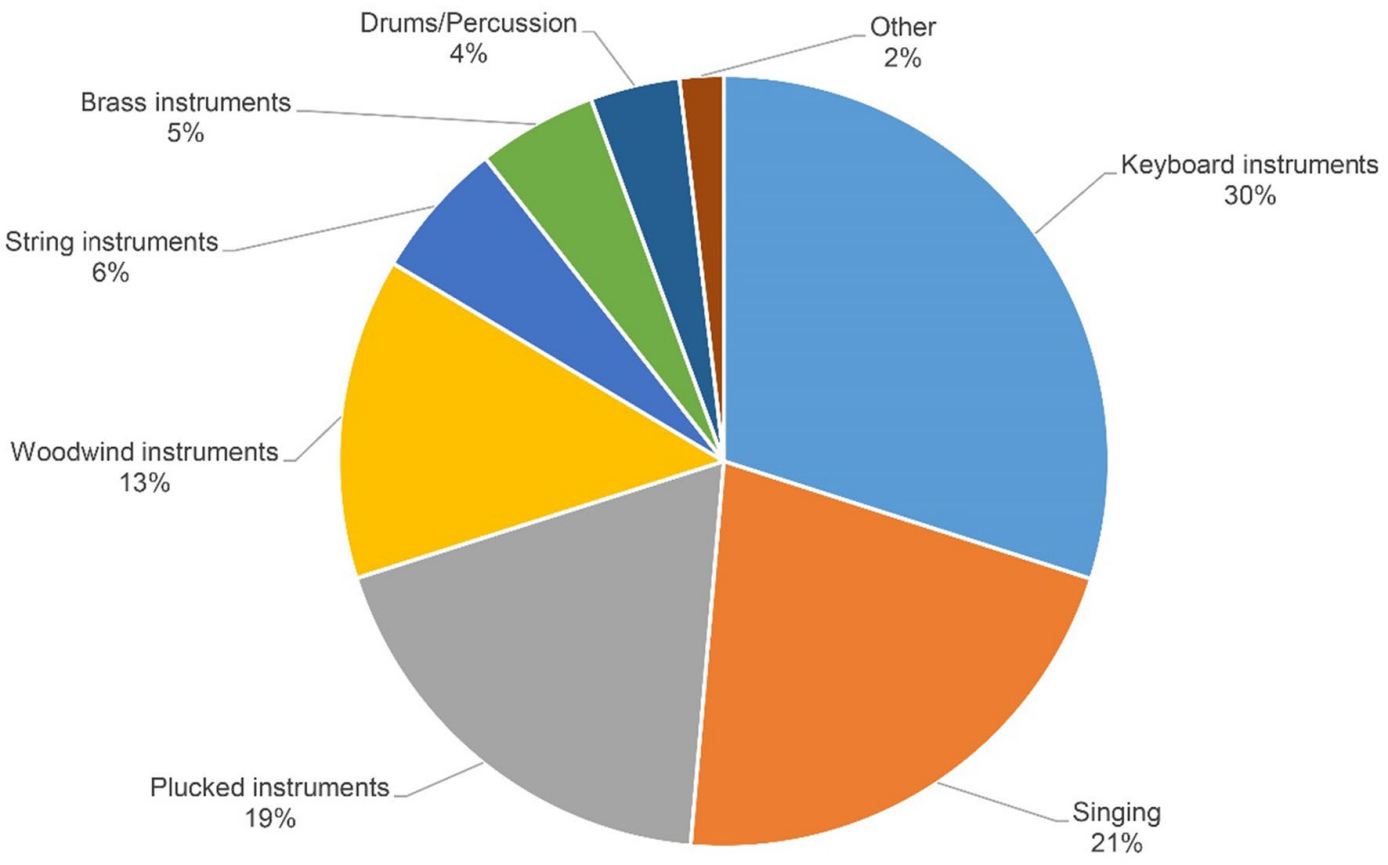
Main instruments of musically active participants (n = 3409).

At the individual peak of MA among all musically active participants, keyboard instruments represented the most frequently played instrument group (30%, n = 1020), followed by singing (21%, n = 731) and plucked instruments (19%, n = 639) (Fig. 2). As shown in Supplemental Table 2, there were some peculiarities in the characteristics of participants depending on the main instrument.

### Music listening

In addition to active music making, music listening was assessed by the MusA questionnaire. In the present study 6533 participants reported that they listened to music in median for 90 min per day (IQR 30.0–150.0). An ANOVA/ANCOVA across tertiles of music listening minutes per day (T1 (Min–Max): 0–50 min/day, T2: 60–120 min/day, T3: 125–1440 min/day) showed trends of linear associations in basic characteristics across the tertiles (Table 3).

**Table 3:** Basic characteristics across tertiles of music listening minutes (minutes/day) (n = 6533, missing information n = 184).

		Tertiles of music listening minutes			p for trend
		T1 (n = 1846)	T2 (n = 2875)	T3 (n = 1812)	
Music listening (minutes/day)	Median (IQR)	30 (10–30)	90 (60–120)	240 (180–360)	
	Min–max	0–50	60–120	125–1440	
Age (years)	Median (IQR)	49.0 (43.0–57.0)	51.0 (43.0–60.0)	52.0 (43.0–63.0)	0.006
Sex					0.66
Men	Percentage (n)	49.2% (908)	50.5% (1453)	49.9% (904)	
Women	Percentage (n)	50.8% (938)	49.5% (1422)	50.1% (908)	
Lifestyle					
Smoking status					< 0.0001
Non-smoker	Percentage (n)	47.6% (861)	44.2% (1248)	41.6% (738)	
Ex-smoker	Percentage (n)	33.7% (609)	34.0% (962)	33.3% (591)	
Smoker	Percentage (n)	18.7% (337)	21.8% (616)	25.1% (444)	
Alcohol consumption (g/day)					
Men	Median (IQR)	8.9 (2.4–19.3)	9.5 (2.9–20.5)	9.1 (2.9–20.6)	0.07
Women	Median (IQR)	3.9 (0.8–10.6)	4.0 (1.2–10.3)	4.1 (1.1–11.3)	0.07
Risky alcohol consumption^a^	Percentage (n)	38.6% (696)	41.6% (1173)	41.4% (736)	0.10
Physical activity (MET-min/week)	Median (IQR)	2880 (1360–5920)	3720 (1720–8160)	4680 (2040–10,440)	< 0.0001
BMI (kg/m^2^)	Median (IQR)	24.3 (22.0–27.0)	25.0 (22.5–28.0)	25.3 (22.8–28.4)	< 0.0001
Underweight (< 18.5 kg/m^2^)	Percentage (n)	1.5% (28)	1.0% (28)	1.2% (21)	< 0.0001
Normal weight (18.5 ≤ to < 25.0 kg/m^2^)	Percentage (n)	55.1% (1016)	48.0% (1377)	45.0% (815)	
Overweight (≥ 25.0 to < 30.0 kg/m^2^)	Percentage (n)	31.1% (573)	36.3% (1040)	36.5% (660)	
Obesity (≥ 30.0 kg/m^2^)	Percentage (n)	12.3% (226)	14.7% (424)	17.3% (313)	
Waist circumference (cm)					
Men	Median (IQR)	91.0 (83.7–99.0)	93.1 (85.2–100.6)	94.4 (86.3–102.7)	< 0.0001
Women	Median (IQR)	79.2 (73.3–88.0)	81.0 (73.8–89.5)	82.0 (74.9–91.2)	0.0001
Monthly equivalised income (€)	Median (IQR)	2150 (1461–3166)	2100 (1452–2833)	1833 (1309–2533)	< 0.0001
Education					< 0.0001
High	Percentage (n)	78.9% (1312)	70.7% (1826)	61.0% (983)	
Medium	Percentage (n)	19.7% (328)	28.2% (728)	37.0% (597)	
Low	Percentage (n)	1.4% (23)	1.1% (28)	2.0% (32)	
Household size					< 0.0001
Single person household	Percentage (n)	24.4% (451)	25.3% (726)	27.6% (500)	
Household with 2 persons	Percentage (n)	39.8% (733)	45.0% (1294)	50.4% (913)	
Household more than 3 persons	Percentage (n)	35.8% (661)	29.7% (855)	22.0% (399)	
Living situation					0.08
Apartment	Percentage (n)	85.5% (1575)	84.1% (2417)	84.6% (1531)	
House	Percentage (n)	14.5% (268)	15.8% (455)	15.2% (276)	
Assisted living	Percentage (n)	0.0% (0)	0.1% (1)	0.2% (4)	
Employment status					< 0.0001
Employment	Percentage (n)	86.0% (1150)	79.7% (2273)	72.3% (1294)	
Unemployed	Percentage (n)	2.7% (49)	3.0% (85)	3.6% (65)	
Retired	Percentage (n)	11.3% (206)	17.3% (495)	24.1% (431)	
Extent of employment					0.63
Full-time (≥ 35h/week)	Percentage (n)	70.2% (1072)	70.5% (1562)	69.0% (862)	
Part-time (< 35h/week)	Percentage (n)	29.8% (456)	29.5% (654)	31.0% (388)	
Other					
Subjective life satisfaction					0.04
9 to completely satisfied (10)	Percentage (n)	34.4% (619)	35.2% (990)	37.3% (660)	
6 to 8	Percentage (n)	51.9% (935)	53.5% (1507)	50.2% (888)	
5	Percentage (n)	6.3% (114)	5.3% (149)	5.8% (104)	
2 to 4	Percentage (n)	6.6% (119)	4.9% (137)	6.1% (108)	
Completely unsatisfied (0) to 1	Percentage (n)	0.8% (15)	1.1% (30)	0.6% (10)	
Subjective health status					0.02
Excellent	Percentage (n)	4.7% (84)	4.7% (133)	4.7% (83)	
Very good	Percentage (n)	38.1% (687)	33.9% (952)	32.4% (573)	
Good	Percentage (n)	49.2% (887)	53.0% (1491)	53.8% (953)	
Less good	Percentage (n)	7.1% (128)	7.9% (222)	8.3% (147)	
Bad	Percentage (n)	0.9% (16)	0.5% (15)	0.8% (14)	
Musically active	Percentage (n)	59.1% (1079)	54.0% (1537)	46.5% (826)	< 0.0001
Cumulative lifetime music-hours (hours)^bc^	Geometric mean (95%-CI)	1003 (745–1343)	1139 (855–1518)	1136 (852–1514)	0.10
Lifetime active music years (years)^bc^	Geometric mean (95%-CI)	6.7 (5.4–8.3)	6.8 (5.6–8.4)	6.4 (5.2–7.9)	0.37
Practice density (hours/active music year)^bc^	Geometric mean (95%-CI)	150 (130–172)	166 (145–191)	178 (155–205)	< 0.0001

Data expressed as median (IQR) or percentage (n), p-values were calculated by chi-square tests or ANOVA/ANCOVA testing for linear trend.

^a^Risky alcohol consumption was defined as AUDIT-C Score > 4 for men, and > 3 for women.

^b^Data expressed as geometric mean (95%-CI), ANCOVA adjusted for sex, age, education, smoking, physical activity, BMI, employment status, monthly equivalized income.

^c^Considering only musically active participants.

We observed that the amount of music listening was associated with higher age, BMI, waist circumference, as well as practice density across the tertiles (Table 3). In contrast, monthly equivalized income and education were inversely related to music listening. Individuals with low music listening time were more frequently non-smokers as compared to the intense listeners (Table 3). In addition, there was a positive association between smaller household size (i.e. single person household, household with 2 persons) with time spent listening to music. However, households with larger numbers of individuals showed lower affinity to music listening. Moreover, participants who were employed tended to spend less time with listening to music (inverse association across tertiles), but contrasting information was observed in unemployed or retired participants (Table 3).

**Figure 3:**
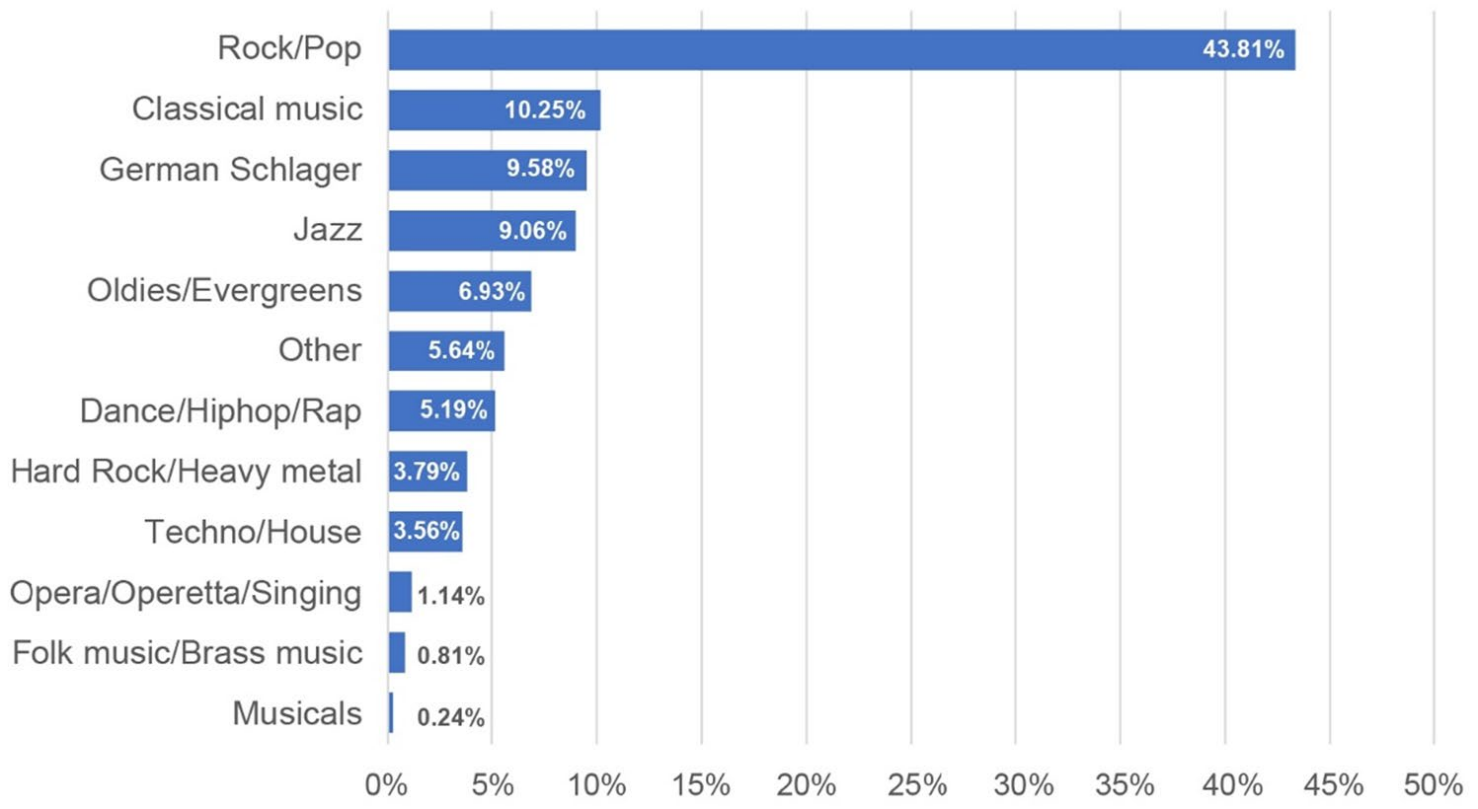
Percentage rates of music genres^a^ most often indicated as favorites (n = 6576, missing information n = 141). ^a^List of music genres predefined in the MusA Questionnaire.

With regard to the most popular music genres, it was found that Rock/Pop (43.8%), Classical music (10.3%) and German Schlager (9.6%) were preferred most often (Fig. 3). As shown in Supplemental Table 6 the preferences for music genres were equally distributed across sex and age with few exceptions. Musicals were more often preferred by women, and Hard rock/Heavy metal, Folk music/Brass music and Techno/House were more often favored by men. Younger people tended to listen to Techno/House, while older participants preferred Opera/Operetta/Singing and German Schlager.

## Discussion

The study presents a comprehensive description of demographic, health- and lifestyle factors associated with active and receptive musical activity in a cohort of over 6000 participants. The findings also take into account the instruments being played, the engagement in singing, and an assessment of preferred music genres when listening to music. The present work focuses on amateur musicians only, professional musicians were excluded from the analyses. These findings are important, because the level of detailed information with regard to different kinds of MA can rarely be found in previous studies. However, this work is aligned with recent developments of inventories to address both active and receptive MA in recent cohort studies on healthy ageing^36^.

In the current sample, about every second person (53%) of the population had been musically active at least once in their life. This proportion is similar to findings from a Danish cohort study with respect to the participating amateur musicians^25^. MA is most common in the first decade of life, but decreases after the age of 20, presumably due to other life tasks such as continued education, starting a profession, or raising a family. It is likely that MA in the early years is at least partially driven by socioeconomic and educational factors, which may disadvantage families with low income and/or education in this regard. However, the importance of MA in childhood and adolescence with respect to cognitive and social-emotional development has been implicated in previous work using data from panel studies^37,38^. These lines of research suggest a greater role of parental education for the amount of MA in children, and a more mixed picture regarding family income. Interestingly, a statistical survey of amateur musicians in Germany noticed that most of those who are musically active at the age of 30 usually remain so into old age^2^. Indeed, also the present study reveals that 75% of the participants who are musically active during the life stage of 31–50 years are still active beyond their 50th birthday. The lifestyle characteristics of musically active individuals showed a trend of drinking more alcohol, being less physically active, and having a lower BMI in comparison to musically inactive individuals. This pattern resembles previous observations concerning social groups that are differentiated by socioeconomic status and education^39,40^. Previous studies demonstrated that playing music instruments may result in physiological activation and energy expenditure^41–43^. Whether or not this activation has an influence on the BMI, has not yet been investigated. In addition, the potential influences of MA on lifestyle, leading to lower BMI, for example, should be a subject of future investigations. Those may also address the question, whether musical activity can help developing social skills and networks with more active lifestyles, especially when it comes to playing music in families^38,44^.

The detailed comparison between groups with lower and higher MA in the current study shows that the latter group accumulated a four-fold greater number of years of musical engagement over the life course and that they had a double practice density.

However, no differences have been observed between the two groups in the majority of lifestyle factors, with the exception of education. Participants with a higher musical engagement had a higher level of education. This underlines the proposed connection between education and MA, a finding that is mirrored in a recent survey on amateur music in Germany. Therein, 25% of teenagers with a higher socio-economic status played music, but only 12% from the lower and 14% from the middle class^2^. In addition, MA of younger individuals seems to depend on the educational status and income of their parents^45^.

The current study allows singers and instrumentalists to be considered independently. The main instruments in the musically active population were keyboards and plucked instruments, a finding that partially mirrors the previously cited report, which also entails a higher prevalence for males playing electronic and brass instruments and females being more active with woodwind instruments^2^. We also note a sex imbalance favoring female singers, which could be expected from previous work on amateur singing in Germany^46^. Interestingly, we further observed a lower education in the group of singers and plucked instruments than in those playing keyboard instruments. However, these results need replication in other population-based samples. Our data also show a trend towards a slightly lower socioeconomic status of the singers compared to the MA group as a whole.

Music listening was found to be associated with greater physical activity, but showed an inverse relationship with socioeconomic indicators. The observation that frequent listening was found more often in individuals with lower socioeconomic could mirror their reduced access to MA^46^. Alternatively, music listening could function as a preferred emotion regulation strategy in this group, whereas a more selective music listening might take place in so-called high brow listeners, i.e. listeners with higher levels of education and socioeconomic status^47^. Finally, enhanced levels of music listening which were found associated with greater physical activity could also reflect its wide use in sports activities like jogging^48^. The question of whether and to what extent music listening is a factor to promote more active lifestyles is of interest due to positive effects on mood, physical performance, perceived exertion and oxygen consumption^49^.

The preferences for music genres in the present study were almost equally distributed across sex and age, with women showing more interest in musicals, whereas men were more interested into Hard Rock and Heavy metal. The development of musical taste is influenced by cultural and social environment as well as age, gender, income, personality style and musical experience, which could partially explain the observed gender patterns^50,51^. However, a more detailed analysis will be required to assess genre preferences in different age groups. Knowledge about such music preferences and, for example, familiarity, may be helpful in the application of music listening as strategy in stress regulation^52^.

A strength of our study is the large sample size and the availability of high-quality data. At the present time the use of the MusA questionnaire provided the most comprehensive data on active and receptive MA. Through its use in the NAKO study center Berlin-Mitte, these data were linked with comprehensive information on demographic, health, and lifestyle characteristics. Nevertheless, the cross-sectional design of the present study does not allow a causal inference. Furthermore, we used data from one study center only. Therein, recruitment takes place across a range of different communities across the city of Berlin. However, the results might be not generalizable to other populations. In addition, the MusA survey was applied prior to the COVID-19 pandemic and continued in the midst of the COVID-19 pandemic when extensive infection prevention measures were implemented (e.g. contact restriction). Therefore, it cannot be excluded that the information provided by the participants regarding their active and receptive MA could have been influenced by these circumstances. As a consequence, MA within the last 12 months (also assessed by the MusA questionnaire) was not considered in the framework of this present study. Our working group addresses this scientific research question in detail elsewhere. We would also like to point out once again that the present paper shows the basic characteristics and that more detailed analyses, e.g. on relationships with mental health and cardiovascular health parameters, are planned in further manuscripts.

In conclusion, the study presents a comprehensive cross-sectional description of demographic, health and lifestyle characteristics associated with active and receptive MA based on data from the NAKO Study Center Berlin-Mitte. These data provide an ideal data basis for future prospective studies on the possible effects of MA as an influencing factor on various health-related variables.

### Supplementary Information

Supplementary Tables.

## Data Availability

The German National Cohort (NAKO) data is not openly available due to data protection measures. However, scientists can apply for data access following the official usage regulations and upon formal request to the NAKO use and access committee (https://transfer.nako.de).
