# Supplementary material for: Musical activity in a subsample of the German National Cohort study
Source: Sci Rep. 2024 Jun 18;14:14069. doi: 10.1038/s41598-024-64773-3 (PMC11189540; doi:10.1038/s41598-024-64773-3)
Supplement: Supplementary file 1 — Supplementary Tables. [file 41598_2024_64773_MOESM1_ESM.docx]

**Supplemental Table 1.** Differences in basic characteristics stratified by main instrument among all musically active participants

|  | **Singing**  **(n=731)** | **All instruments**  **(n=2614)** | **Keyboard instruments (n=1020)** | **Plucked instruments (n=638)** |
| --- | --- | --- | --- | --- |
| Cumulative lifetime music-hours (hours) ^a^ | 1147 (847-1553) | 1285 (923-1788) | 1217 (905-1637) | 1213 (897-1641) |
| Lifetime active music years (years) ^a^ | 7.8 (6.3-9.8) | 7.1 (5.6-9.0) | 6.8 (5.5-8.5) | 6.9 (5.5-8.6) |
| Practice density (hours/ active music year) ^a^ | 147 (126-170) | 182 (154-214) | 178 (154-206) | 176 (152-204) |
| Musical activity for entire life | 17.6% (129) | 13.0% (340) | 13.0% (133) | 12.5% (80) |
| Age (years) | 50.0 (42.0-59.0) | 48.0 (41.0-56.0) | 48.0 (40.0-58.0) | 49.0 (42.0-55.0) |
| Sex |  |  |  |  |
| Men | 29.1% (213) | 48.2% (1260) | 41.4% (422) | 57.8% (369) |
| Women | 70.9% (518) | 51.8% (1354) | 58.6% (598) | 42.2% (269) |
| Lifestyle |  |  |  |  |
| Smoking status |  |  |  |  |
| Non-smoker | 45.3% (325) | 43.8% (1130) | 47.0% (472) | 39.2% (247) |
| Ex-smoker | 33.3% (239) | 35.4% (911) | 33.0% (332) | 41.3% (260) |
| Smoker | 21.4% (153) | 20.8% (535) | 20.0% (201) | 19.5% (123) |
| Alcohol consumption (g/ d) |  |  |  |  |
| Men | 9.7 (2.6-18.3) | 9.8 (3.2-20.0) | 9.6 (3.0-18.4) | 10.5 (3.8-19.9) |
| Women | 4.1 (1.3-11.6) | 4.5 (1.4-12.3) | 4.5 (1.5-13.4) | 4.7 (1.1-11.8) |
| Risky alcohol consumption | 40.7% (292) | 43.6% (1123) | 42.7% (429) | 44.5% (280) |
| Physical activity (MET-min/ week) | 3480 (1800-7200) | 3360 (1680-6920) | 3240 (1520-6920) | 3320 (1680-6240) |
| BMI (kg/ m^2^) | 24.3 (22.0-27.5) | 24.3 (21.9-26.8) | 24.2 (21.7-26.8) | 24.4 (22.2-27.0) |
| Waist circumference (cm) |  |  |  |  |
| Men | 92.6 (86.8-99.5) | 90.4 (83.0-97.7) | 90.6 (83.3-97.5) | 90.4 (83.4-97.3) |
| Women | 81.0 (74.0-88.7) | 78.6 (72.9-87.3) | 79.0 (72.7-87.8) | 80.5 (73.9-88.2) |
| Monthly equivalised income (€) | 2033 (1433-2833) | 2125.0 (1433-2916) | 2150 (1461-3050) | 2125 (1461-2916) |
| Education |  |  |  |  |
| High | 77.2% (496) | 80.3% (1858) | 82.5% (749) | 78.2% (438) |
| Medium | 22.2% (143) | 19.1% (443) | 17.1% (155) | 21.1% (118) |
| Low | 0.6% (4) | 0.6% (13) | 0.4% (4) | 0.7% (4) |
| Household size |  |  |  |  |
| Single person household | 28.0% (205) | 24.3% (636) | 23.5% (240) | 22.7% (145) |
| Household with 2 persons | 43.0% (314) | 41.3% (1078) | 42.5% (433) | 41.5% (265) |
| Household more than 3 persons | 29.0% (212) | 34.4% (900) | 34.0% (347) | 35.8% (228) |
| Living situation |  |  |  |  |
| Apartment | 88.0% (643) | 86.0% (2248) | 86.4% (881) | 84.5% (539) |
| House | 12.0% (88) | 13.9% (363) | 13.6% (139) | 15.5% (71) |
| Assisted living | 0.0% (0) | 0.1% (2) | 0.0% (0) | 0.0% (0) |
| Employment status |  |  |  |  |
| Employment | 81.7% (594) | 83.3% (2161) | 81.0% (818) | 85.7% (544) |
| Unemployed | 2.9% (21) | 2.6% (67) | 2.7% (27) | 3.1% (20) |
| Retired | 15.4% (112) | 14.1% (366) | 16.3% (165) | 11.2% (71) |
| Extent of employment |  |  |  |  |
| Full-time (≥35h/ week) | 64.3% (375) | 67.2% (1402) | 65.5% (516) | 68.5% (361) |
| Part-time (<35h/ week) | 35.7% (208) | 32.8% (683) | 34.5% (272) | 31.5% (166) |
| Other |  |  |  |  |
| Subjective life satisfaction |  |  |  |  |
| 9 to completely satisfied (10) | 31.0% (221) | 34.0% (872) | 35.8% (361) | 31.6% (198) |
| 6 to 8 | 57.0% (407) | 53.5% (1375) | 52.5% (528) | 54.9% (344) |
| 5 | 5.9% (42) | 5.9% (152) | 5.9% (59) | 5.0% (31) |
| 2 to 4 | 5.7% (41) | 5.8% (149) | 5.0% (50) | 7.4% (46) |
| Completely unsatisfied (0) to 1 | 0.4% (3) | 0.8% (21) | 0.8% (8) | 1.1% (7) |
| Subjective health status |  |  |  |  |
| Excellent | 4.5% (32) | 5.8% (150) | 6.0% (60) | 5.4% (34) |
| Very good | 33.9% (242) | 39.4% (1013) | 39.8% (400) | 39.3% (246) |
| Good | 53.6% (383) | 47.8% (1229) | 47.9% (482) | 47.3% (296) |
| Less good | 7.7% (55) | 6.4% (163) | 5.9% (60) | 7.4% (46) |
| Bad | 0.3% (2) | 0.6% (14) | 0.4% (4) | 0.6% (4) |

Data expressed as median (IQR) or percentage (n), ^a^ Data expressed as geometric mean (95%-CI), adjusted for sex, age, education, smoking, physical activity, BMI, employment status, monthly equivalized income

**Supplemental Table 1 (continued).** Differences in basic characteristics stratified by main instrument among all musically active participants

|  | **Woodwind instruments (n=459)** | **String instruments (n=198)** | **Brass instruments (n=171)** | **Drums/ Percussion**  **(n=128)** |
| --- | --- | --- | --- | --- |
| Cumulative lifetime music-hours (hours) ^a^ | 865 (628-1192) | 1673 (1179-2373) | 1233 (850-1788) | 1205 (829-1751) |
| Lifetime active music years (years) ^a^ | 6.1 (4.8-7.7) | 8.6 (6.7-11.1) | 6.7 (5.1-8.8) | 6.7 (5.1-8.8) |
| Practice density (hours/ active music year) ^a^ | 142 (122-166) | 195 (164-231) | 184 (153-220) | 181 (150-217) |
| Musical activity for entire life | 9.8% (45) | 20.7% (41) | 13.5% (23) | 14.1% (18) |
| Age (years) | 48.0 (42.0-56.0) | 47.0 (39.0-55.0) | 49.0 (40.0-58.0) | 45.5 (34.0-53.0) |
| Sex |  |  |  |  |
| Men | 33.5% (154) | 50.0% (99) | 70.2% (120) | 75.0% (96) |
| Women | 66.5% (305) | 50.0% (99) | 29.8% (51) | 25.0% (32) |
| Lifestyle |  |  |  |  |
| Smoking status |  |  |  |  |
| Non-smoker | 45.8% (207) | 50.2% (98) | 37.3% (63) | 34.4% (43) |
| Ex-smoker | 33.9% (153) | 30.3% (59) | 39.1% (66) | 32.8% (41) |
| Smoker | 20.3% (92) | 19.5% (38) | 23.6% (40) | 32.8% (41) |
| Alcohol consumption (g/ d) |  |  |  |  |
| Men | 10.4 (3.0-20.0) | 12.0 (3.1-20.1) | 8.2 (2.7-21.9) | 9.7 (3.5-22.3) |
| Women | 4.5 (1.3-11.9) | 4.9 (1.6-10.8) | 4.6 (2.0-12.6) | 1.5 (0.5-8.9) |
| Risky alcohol consumption | 40.8% (185) | 45.1% (88) | 48.5% (81) | 48.0% (60) |
| Physical activity (MET-min/ week) | 3040 (1560-6240) | 3360 (1600-6480) | 4200 (1920-9600) | 4000 (2340-9080) |
| BMI (kg/ m^2^) | 24.1 (21.6-26.8) | 23.8 (21.9-26.1) | 24.6 (21.7-27.3) | 24.6 (22.0-26.6) |
| Waist circumference (cm) |  |  |  |  |
| Men | 91.0 (83.5-98.0) | 90.4 (83.3-98.5) | 91.9 (83.2-100.0) | 88.2 (80.7-96.2) |
| Women | 78.7 (73.6-88.6) | 77.4 (71.2-83.5) | 74.3 (71.3-81.1) | 76.5 (70.9-88.9) |
| Monthly equivalised income (€) | 2125 (1527-2875) | 2150 (1433-3400) | 2033 (1433-2833) | 1816 (1343-2491) |
| Education |  |  |  |  |
| High | 79.4% (316) | 85.8% (157) | 75.7% (115) | 73.5% (83) |
| Medium | 20.6% (82) | 13.6% (25) | 24.3% (37) | 23.0% (26) |
| Low | 0.0% (0) | 0.6% (1) | 0.0% (0) | 3.5% (4) |
| Household size |  |  |  |  |
| Single person household | 28.3% (130) | 25.8% (51) | 18.1% (31) | 30.5% (39) |
| Household with 2 persons | 38.6% (177) | 34.3% (68) | 52.6% (90) | 35.1% (45) |
| Household more than 3 persons | 33.1% (152) | 39.9% (79) | 29.3% (50) | 34.4% (44) |
| Living situation |  |  |  |  |
| Apartment | 84.5% (388) | 88.8% (175) | 88.9% (152) | 88.3% (113) |
| House | 15.5% (71) | 10.7% (21) | 11.1% (19) | 10.9% (14) |
| Assisted living | 0.0% (0) | 0.5% (1) | 0.0% (0) | 0.8% (1) |
| Employment status |  |  |  |  |
| Employment | 84.6% (386) | 85.7% (168) | 87.9% (140) | 83.3% (105) |
| Unemployed | 1.8% (8) | 3.6% (7) | 0.6% (1) | 3.2% (4) |
| Retired | 13.6% (62) | 10.7% (21) | 17.5% (30) | 13.5% (17) |
| Extent of employment |  |  |  |  |
| Full-time (≥35h/ week) | 67.5% (251) | 65.6% (105) | 73.0% (100) | 68.3% (69) |
| Part-time (<35h/ week) | 32.5% (121) | 34.4% (55) | 27.0% (37) | 31.7% (32) |
| Other |  |  |  |  |
| Subjective life satisfaction |  |  |  |  |
| 9 to completely satisfied (10) | 33.2% (150) | 35.6% (69) | 34.4% (57) | 29.6% (37) |
| 6 to 8 | 53.3% (241) | 51.5% (100) | 53.0% (88) | 59.2% (74) |
| 5 | 8.0% (36) | 6.2% (12) | 4.8% (8) | 4.8% (6) |
| 2 to 4 | 4.6% (21) | 5.7% (11) | 7.8% (13) | 6.4% (8) |
| Completely unsatisfied (0) to 1 | 0.9% (4) | 1.0% (2) | 0.0% (0) | 0.0% (0) |
| Subjective health status |  |  |  |  |
| Excellent | 5.3% (24) | 6.7% (13) | 6.0% (10) | 7.2% (9) |
| Very good | 39.4% (178) | 40.2% (78) | 38.0% (63) | 38.4% (48) |
| Good | 48.2% (218) | 47.4% (92) | 50.0% (83) | 46.4% (58) |
| Less good | 6.4% (29) | 5.2% (10) | 6.0% (10) | 6.4% (8) |
| Bad | 0.7% (3) | 0.5% (1) | 0.0% (0) | 1.6% (2) |

Data expressed as median (IQR) or percentage (n), ^a^ Data expressed as geometric mean (95%-CI), adjusted for sex, age, education, smoking, BMI, physical activity, employment status, monthly equivalized income

**Supplemental Table 2.** Basic characteristics according to musical activity stratified sex

|  | **No musical active men (n=1750) ^b^** | **Musical active**  **men (n=1542) ^a^** | **p-value** | **No musical active women (n=1359) ^b^** | **Musical active women (n=1970) ^a^** | **p-value** |
| --- | --- | --- | --- | --- | --- | --- |
| Age (years) | 52.0 (45.0-62.0) | 49.0 (41.0-57.0) | <0.0001 | 53.0 (45.0-62.0) | 49.0 (41.0-58.0) | <0.0001 |
| Lifestyle |  |  |  |  |  |  |
| Smoking status |  |  | 0.13 |  |  | 0.007 |
| Non-smoker | 44.3% (761) | 40.8% (620) |  | 47.0% (622) | 46.0% (891) |  |
| Ex-smoker | 34.4% (590) | 36.1% (549) |  | 29.4% (389) | 34.0% (658) |  |
| Smoker | 21.3% (366) | 23.1% (350) |  | 23.6% (312) | 20.0% (387) |  |
| Risky alcohol consumption ^c^ | 41.4% (711) | 45.7% (694) | 0.01 | 33.7% (445) | 40.0% (773) | 0.0003 |
| Physical activity (MET-min/ week) | 4200 (1760-9440) | 3360 (1680-7000) | <0.0001 | 4080 (1680-9120) | 3360 (1680-7200) | 0.0017 |
| BMI (kg/ m^2^) | 26.3 (23.8-29.1) | 25.0 (22.9-27.5) | <0.0001 | 24.7 (22.1-28.2) | 24.5 (21.4-26.5) | <0.0001 |
| Underweight (< 18.5 kg/ m2) | 0.4% (7) | 0.6% (9) | <0.0001 | 1.1% (15) | 2.6% (51) | <0.0001 |
| Normal weight (18.5 ≤ to < 25.0 kg/ m^2^) | 35.5% (620) | 48.4% (746) |  | 51.2% (695) | 60.9% (1196) |  |
| Overweight (≥ 25.0 to < 30.0 kg/ m^2^) | 44.3% (773) | 40.5% (625) |  | 29.6% (402) | 24.8% (486) |  |
| Obesity (≥ 30.0 kg/ m^2^) | 19.8% (347) | 10.5% (162) |  | 18.1% (245) | 11.7% (231) |  |
| Monthly equivalized income (€) | 2111 (1433-2833) | 2267 (1583-3167) | <0.0001 | 1800 (1375-2533) | 2033 (1433-2812) | <0.0001 |
| Education |  |  | <0.0001 |  |  | <0.0001 |
| High | 63.7% (1036) | 80.4% (1100) |  | 57.2% (695) | 77.9% (1349) |  |
| Medium | 34.4% (553) | 18.8% (258) |  | 40.4% (491) | 21.6% (373) |  |
| Low | 1.9% (31) | 0.8% (11) |  | 2.4% (29) | 0.5% (9) |  |
| Household size |  |  | <0.0001 |  |  | <0.0001 |
| Single person household | 25.6% (448) | 24.1% (371) |  | 26.3% (357) | 26.7% (527) |  |
| Household with 2 persons | 46.9% (820) | 41.2% (636) |  | 51.7% (702) | 42.0% (826) |  |
| Household more than 3 persons | 27.5% (482) | 34.7% (535) |  | 22.1% (300) | 31.3% (1151) |  |
| Living situation |  |  | 0.0002 |  |  | 0.002 |
| Apartment | 81.7% (1428) | 85.0% (1309) |  | 83.5% (1134) | 87.7% (1725) |  |
| House | 18.2% (319) | 14.9% (230) |  | 16.4% (223) | 12.3% (242) |  |
| Assisted living | 0.1% (2) | 0.1% (2) |  | 0.1% (1) | 0.0% (0) |  |
| Employment status |  |  | <0.0001 |  |  | <0.0001 |
| Employment | 77.5% (1343) | 83.3% (1272) |  | 72.2% (965) | 82.2% (1609) |  |
| Unemployed | 3.5% (62) | 2.6% (40) |  | 3.1% (42) | 2.9% (57) |  |
| Retired | 19.0% (329) | 14.1% (216) |  | 24.7% (330) | 14.9% (292) |  |
| Extent of employment |  |  | <0.0001 |  |  | 0.14 |
| Full-time (≥35h/ week) | 81.8% (1077) | 75.5% (936) |  | 62.6% (583) | 59.7% (926) |  |
| Part-time (<35h/ week) | 18.2% (239) | 25.5% (304) |  | 37.4% (348) | 40.3% (626) |  |
| Other |  |  |  |  |  |  |
| Subjective life satisfaction |  |  | 0.01 |  |  | 0.07 |
| 9 to completely satisfied (10) | 39.2% (669) | 33.1% (503) |  | 36.9% (487) | 33.4% (642) |  |
| 6 to 8 | 49.9% (853) | 54.4% (827) |  | 50.3% (664) | 53.7% (1034) |  |
| 5 | 5.1% (57) | 5.4% (82) |  | 6.0% (79) | 6.3% (121) |  |
| 2 to 4 | 5.0% (86) | 6.0% (91) |  | 5.7% (75) | 6.1% (118) |  |
| Completely unsatisfied (0) to 1 | 0.8% (14) | 1.1% (16) |  | 1.1% (15) | 0.5% (10) |  |
| Subjective health status |  |  | <0.0001 |  |  | <0.0001 |
| Excellent | 3.9% (68) | 6.5% (98) |  | 3.8% (50) | 2.6% (51) |  |
| Very good | 32.7% (558) | 38.9% (591) |  | 28.8% (380) | 60.9% (1196) |  |
| Good | 54.6% (933) | 47.6% (723) |  | 56.2% (742) | 24.8% (486) |  |
| Less good | 8.1% (138) | 6.3% (96) |  | 10.1% (133) | 11.7% (231) |  |
| Bad | 0.7% (12) | 0.7% (11) |  | 1.1% (15) | 0.0% (0) |  |

Data expressed as median (IQR) or percentage (n), ^a^ Participants count as non-musically active if they have not played an instrument or sung at any stage of life. ^b^ Participants count as musically active if they have played an instrument or sung at a single or multiple stages of life. ^c^ Risky alcohol consumption was defined as AUDIT-C Score >4 for men, and >3 for women

**Supplemental Table 3.** Extent and practice time of musical activities in individual life stages

| Life stage |  | n | Median (IQR) |
| --- | --- | --- | --- |
| ≤10 years | Years of musical activity in life stage (years) | 1871 | 3 (2-4) |
|  | Weekly active music hours in life stage (hours/ week) | 1871 | 2 (2-4) |
| 11-20 years | Years of musical activity in life stage (years) | 2365 | 5 (3-8) |
|  | Weekly active music hours in life stage (hours / week) | 2365 | 3 (2-5) |
| 21-30 years | Years of musical activity in life stage (years) | 943 | 5 (3-9) |
|  | Weekly active music hours in life stage (hours/ week) | 943 | 3 (2-5) |
| 31-50 years | Years of musical activity in life stage (years) | 878 | 7 (3-12) |
|  | Weekly active music hours in life stage (hours/ week) | 878 | 2 (2-4) |
| > 50 years | Years of musical activity in life stage (years) | 417 | 4 (2-9) |
|  | Weekly active music hours in life stage (hours/ week) | 417 | 3 (2-4) |

Detailed list of years of musical activity (years) and weekly practice hours (hours/ week) in five life stages

**Supplemental Table 4. Linear regression models on the association between cumulative lifetime music-hours (log-transformed) and potential determinants**

|  | Model 1 (unadjusted) | | |
| --- | --- | --- | --- |
| Predefined determinates | n | β-coefficients (95%-CI) | p-value |
| Sex | 6717 | 0.65 (0.48 to 0.82) | <0.0001 |
| Age (log-transformed) | 6717 | -2.17 (-2.47 to -1.86) | <0.0001 |
| Physical activity (log-transformed) | 6022 | -0.29 (-0.37 to -0.21) | <0.0001 |
| BMI (log-transformed) | 6704 | -3.54 (-4.04 to -3.03) | <0.0001 |
| Monthly equivalized income (log-transformed) | 6417 | 0.28 (0.13 to 0.43) | 0.0003 |
| Smoking status | 6717 |  |  |
| Intercept (Nonsmoker) |  | 3.18 (3.05 to 3.31) | <0.0001 |
| Ex-smoker |  | 0.20 (0.00 to 0.39) | 0.049 |
| Smoker |  | -0.05 (-0.27 to 0.18) | 0.68 |
| Education | 6717 |  |  |
| Intercept (Low) |  | 3.28 (3.03 to 3.52) | <0.0001 |
| Medium |  | -1.16 (-1.45 to -0.86) | <0.0001 |
| High |  | 0.40 (0.12 to 0.66) | 0.0036 |
| Employment status | 6717 |  |  |
| Intercept (Retired) |  | 2.46 (2.26 to 2.66) | <0.0001 |
| Employed |  | 0.98 (0.78 to 1.20) | <0.0001 |
| Unemployed |  | 0.36 (-0.16 to 0.88) | 0.18 |

**Supplemental Table 5. Multiple linear regression model on the association between cumulative lifetime music-hours (log-transformed) and potential determinants mutually adjusted**

|  | Model 2 (mutually adjusted) n=5769 | |
| --- | --- | --- |
|  | β-coefficients (95%-CI) | p-value |
| Intercept | 15.30 (12.86 to 17.74) | <0.0001 |
| Sex | 0.55 (0.37 to 0.73) | <0.0001 |
| Age (log-transformed) | -1.72 (-2.08 to -1.35) | <0.0001 |
| Physical activity (log-transformed) | -0.13 (-0.21 to -0.05) | 0.0015 |
| BMI (log-transformed) | -2.08 (-2.66 to -1.51) | <0.0001 |
| Monthly equivalized income (log-transformed) | 0.17 (-0.002 to 0.34) | 0.05 |
| Ex-smoker | 0.47 (0.26 to 0.67) | <0.0001 |
| Smoker | 0.11 (-0.12 to 0.35) | 0.35 |
| Medium Education | -1.14 (-1.46 to -0.83) | <0.0001 |
| High Education | 0.26 (-0.03 to 0.55) | 0.08 |
| Employed | 0.34 (0.08 to 0.59) | 0.009 |
| Unemployed | 0.33 (-0.22 to 0.89) | 0.24 |

**Supplemental Table 6. Preferences for music genres (n=6576) across sex and age**

|  | Men | Women | Age |
| --- | --- | --- | --- |
|  | % (n) | % (n) | Median (IQR) |
| Rock/ Pop | 48.6% (1401) | 51.4% (1480) | 49.0 (42.0-56.0) |
| Classical music | 42.9% (289) | 57.1% (385) | 57.0 (48.0-65.0) |
| German Schlager | 45.1% (284) | 54.9% (346) | 61.0 (52.0-66.0) |
| Jazz | 51.2% (305) | 48.8% (291) | 52.0 (44.0-59.0) |
| Oldies/ Evergreens | 44.1% (201) | 55.9% (255) | 60.0 (52.0-65.0) |
| Other | 51.5% (191) | 48.5% (180) | 47.0 (41.0-54.0) |
| Dance/ Hip Hop/ Rap | 50.7% (173) | 49.3% (168) | 41.0 (29.0-49.0) |
| Hard Rock/ Heavy Metal | 76.3% (190) | 23.7% (59) | 47.0 (39.0-53.0) |
| Techno/ House | 65.4% (153) | 34.6% (81) | 37.0 (28.0-47.0) |
| Folk music/Brass music | 66.0% (35) | 34.0% (18) | 58.0 (48.0-66.0) |
| Opera/ Operetta/ Singing | 48.0% (36) | 52.0% (39) | 62.0 (53.0-67.0) |
| Musicals | 37.5% (6) | 62.5% (10) | 55.0 (50.0-62.0) |

List of music genres predefined in the MusA Questionnaire
